# Pseudohypoparathyroidism type 1a due to a novel mutation in the *GNAS* gene

**DOI:** 10.1111/cen.12953

**Published:** 2015-10-26

**Authors:** Manuel C. Lemos, Paul T. Christie, Dírcea Rodrigues, Rajesh V. Thakker

**Affiliations:** ^1^ CICS‐UBI, Health Sciences Research CentreUniversity of Beira InteriorCovilhãPortugal; ^2^ Academic Endocrine UnitRadcliffe Department of MedicineOxford Centre for Diabetes, Endocrinology and Metabolism (OCDEM)Churchill HospitalUniversity of OxfordOxfordUK; ^3^ Endocrinology, Diabetes and Metabolism ServiceUniversity Hospital of CoimbraCoimbraPortugal

## Financial disclosure

The authors have nothing to disclose.

Dear Editor,

Pseudohypoparathyroidism type 1a (PHP1a) (OMIM #103580) is characterized by hypocalcaemia and hyperphosphataemia due to parathyroid hormone (PTH) resistance, associated with features of Albright's Hereditary Osteodystrophy (AHO) which include short stature, obesity, subcutaneous calcifications and brachydactyly.^1^ PHP1a is caused by heterozygous germline mutations of the alpha subunit of the stimulatory form of the GTP‐binding protein (Gs‐alpha), which is a downstream signalling protein of the PTH receptor and of other G protein‐coupled hormone receptors.^1^ Gs‐alpha is encoded by the *GNAS* gene (chromosome 20q13.3), which is a complex imprinted locus that also produces additional coding and noncoding transcripts through the use of alternative promoters and alternative splicing, in a tissue‐specific manner.^2^ PHP1a results from maternally inherited loss‐of‐function mutations of Gs‐alpha, but paternally inherited mutations usually result in pseudopseudohypoparathyroidism (PPHP), which is characterized by the presence of AHO without hormone resistance. Thus, PHP1a and PPHP, which are inherited as autosomal dominant disorders with parental imprinting, are frequently found in the same kindred.

**Figure 1:**
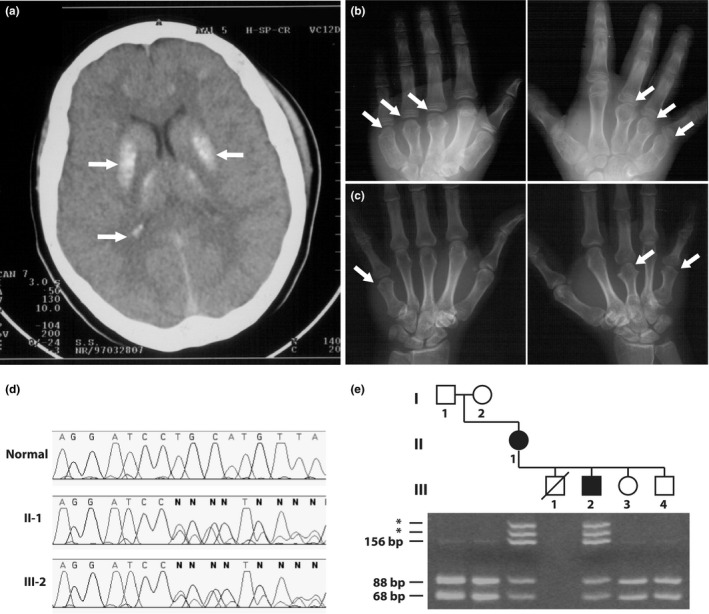
(a) Head computed tomography (CT) scan of the affected child showing calcification of basal ganglia and of other dispersed regions of the brain (arrows). (b) Radiograph showing shortening bilaterally of the 3rd, 4th and 5th metacarpals (arrows) in the affected child, typical of Albright's osteodystrophy. (c) Shortening of the left 5th, and right 3rd and 5th metacarpals in the mother who has pseudopseudohypoparathyroidism (PPHP). (d) DNA sequence analysis of both mother (II‐1) and child (III‐2) revealed a frameshift that resulted from a heterozygous 2‐base pair (bp) deletion at codon 63 (c.188_189delTG). The mutation nomenclature was based on the *GNAS* cDNA reference sequence (GenBank Accession number NM_000516.4). (e) This 2‐bp deletion resulted in the loss of an *Nsp*I restriction enzyme site in the mutant sequence, and this facilitated the screening of the mutation in other family members. Electrophoresis of *Nsp*I digested PCR fragments on an 8% polyacrylamide gel showed that one product of 156 bp was obtained from the mutant sequence (additional heteroduplex products are indicated by asterisks), but two products of 88 and 68 bp were obtained from the wild‐type normal sequence. The affected individuals are heterozygous, and the unaffected are homozygous for the wild‐type allele. Individuals are represented as males (squares), females (circles), unaffected (open symbol), affected (filled symbol) and deceased (oblique line through symbol). Individual III‐1 was reported to share a similar phenotype as III‐2 by his relatives.

We identified a kindred with PHP1a/PPHP and investigated the patients for the underlying molecular abnormality. The index case was an 11‐year‐old Portuguese boy that presented with seizures due to hypocalcaemia. Serum concentration of calcium was 1·65 mmol/l (normal: 2·0–2·6 mmol/l), phosphate 3·6 mmol/l (normal: 0·9–1·5 mmol/l) and PTH 607 ng/l (normal: 9–55). Height and weight were on the 50th percentile and 90th percentile, respectively. He had subcutaneous and intracranial calcifications (Fig. 1a); brachydactyly, which was due to shortened metacarpals and metatarsals (Fig. 1b); and learning disability. He also had abnormal thyroid function tests with serum concentrations of TSH 4·8 mIU/l (normal: 0·3–3·0), free T4 6·4 pmol/l (normal: 7·7–23·2) and free T3 6·6 pmol/l (normal: 3·4–8·4). His mother had short stature (3rd percentile) and brachydactyly (Fig. 1c), but no biochemical abnormalities. Family history also included an older sibling with a similar phenotype, who had died due to an unknown cause, at age 7 years, and two younger unaffected siblings.

Venous blood samples were obtained after informed consent, from the index case, the mother, two siblings and the maternal grandparents. Leucocyte DNA was extracted and used with appropriate PCR primers to amplify exons 1–13 of the *GNAS* gene, utilizing conditions previously described.^3^ Bidirectional sequencing of the PCR products was carried out by the use of the same primers, and an automated capillary sequencer (GenomeLab GeXP System, Beckman Coulter, Fullerton, CA, USA).

A novel heterozygous 2‐base pair (bp) deletion in exon 2 (c.188_189delTG) was found in the proband and his mother (Fig. 1d). The deletion of this dinucleotide sequence is predicted to cause a frameshift, with the incorporation of two missense amino acids, followed by a premature stop codon (TAA) in the new frame at codon 65. The mutation resulted in the loss of an *Nsp*I restriction endonuclease site and this was used to confirm its presence and to assess the other family members (Fig. 1e). The mutation was absent in the unaffected family members, including the maternal grandparents, thereby indicating that the mother (individual II‐2 in Fig. 1e) either has a *de novo* mutation involving the paternal allele or that her father has undetected mosaicism. In addition, an analysis of the DNA from 55 unrelated normal individuals (110 alleles) confirmed the absence of this DNA sequence abnormality (data not shown). The different clinical presentation in the son (PHP1a) and mother (PPHP), who harbour the same mutation, can be explained by the characteristic mode of inheritance, which is autosomal dominant with parental imprinting of hormone resistance.

The identification of the causative mutation in the index case may be useful for screening other family members in order to avoid late or misdiagnosis, as probably occurred with individual III‐1 (Fig. 1e). Age of onset of the hormone resistance is quite variable among mutation‐positive individuals and can be delayed for several years. This latency of PTH resistance in patients with PHP1a has been attributed to a gradual development of paternal Gs‐alpha silencing in target tissues.^4^ Therefore, genetic screening of family members can be useful for presymptomatic diagnosis.

There are over 340 reported kindreds with PHP1a/PPHP due to a *GNAS* mutation,^5^ and these are scattered across the 13 exons that encode Gs‐alpha, with no known genotype–phenotype correlation. However, exons 2 and 3 are the least affected in these disorders. To date, only three other mutations of exon 2, one missense and two insertions, have been reported.^5^ Therefore, the 2‐bp deletion in exon 2 identified by the present study is unusual and further expands the spectrum of known *GNAS* mutations associated with these complex disorders.
